# Is Cronbach’s alpha sufficient for assessing the reliability of the OSCE for an internal medicine course?

**DOI:** 10.1186/s13104-015-1533-x

**Published:** 2015-10-19

**Authors:** Aisha M. Al-Osail, Mona H. Al-Sheikh, Emad M. Al-Osail, Mohannad A. Al-Ghamdi, Abdulaziz M. Al-Hawas, Abdullah S. Al-Bahussain, Ahmed A. Al-Dajani

**Affiliations:** University of Dammam, Prince Saud bin Fahd Street, PO Box 3669, Khobar, 31952 Saudi Arabia; University of Dammam, PO Box 2435, Dammam, 31451 Saudi Arabia

**Keywords:** OSCE, Cronbach’s alpha, Reliability, Validity, Spearman’s rank correlation, R2 coefficient, Pearson’s correlation

## Abstract

**Background:**

The number of medical students accepted into medical programs is increasing, which has made the traditional long/short case style of examination difficult to conduct. At Dammam University, the program is shifting to the use of the Objective Structural Clinical Examination (OSCE), which may solve some of these difficulties, including issues with reliability, validity index and exam duration.

**Results:**

A pilot study was conducted over one semester. A total of 207 examinees in three groups took the OSCE and written exams. The OSCE consisted of 18 clinical stations and required 3–4.3 h/day. The written exam contained 80 multiple-choice questions. The Cronbach’s alpha for each group was 0.7, 0.8, and 0.9. Correlations for all stations ranged from 0.7 to 0.8, which indicated good stability and internal consistency with minor differences in the progression of the indexes. The reliability of the written exam was 0.79, and the validity of the OSCE was 0.63, as assessed using Pearson’s correlation.

**Conclusion:**

No single reliability index can be considered a perfect assessment tool to solve this issue. Thus, at least two to three indexes should be used to ensure the reliability of the OSCE.

## Background

Harden and Gleeson implemented the first Objective Structural Clinical Examination (OSCE) as a new examination with sufficient reliability and validity, making the assessment of students more scientific, reliable and valid for both the faculty and examinees [1]. With an increasing number of medical students being accepted into programs worldwide, it has become difficult to assess them in a proper and fair manner using the old traditional style (long and short cases). This is especially true for multi-system courses, such as internal medicine, pediatrics and surgery, where the evaluation of students must include all systems and cover all parts of the assessment areas. Many reliability index measures have been used for the OSCE, including Cronbach’s alpha, Spearman’s rank correlation, and R2 coefficient determinants. All these indexes have been used because no single tool has been considered precise enough. Cronbach’s alpha was created to measure the internal consistency of the exams [2–4]. Although it is considered a good index for station stability, it has some disadvantages: The measure is affected by exam time and dimensionality. As the duration increases, reliability will increase [3, 5, 6]. Therefore, the index measures the stability of the stations (which demonstrates the difference in student performance at each station) but not the internal consistency (which describes the extent to which all the items in a test measure the same concept or constructs). Unfortunately, there are no reports about this is in the OSCE, but there was a report about the effects of different days on the validity of the test [7]. Spearman’s rank correlation coefficient is used to assess the strength and direction of a relationship between two variables or to identify and test the strength of a relationship between two sets of data. Although it has been used in many studies, it has disadvantages [8]: It quantifies only the strength of the linear relationship and highly sensitive to extreme values. The R2 coefficient is a measure of the proportional change in the dependent variable (in our case, the checklist score) compared to changes in the independent variable (the global grade). It is a marker of internal consistency [6–14], but the index is imperfect; if the examiner makes the checklist score correspond to the global score, which means the students did all the items in the checklist, the global score would be a clear pass and vice versa. This would result in false inflation of the R2 because the global rating would score the student’s confidence, organization and professional application of clinical skills, which might not be included in the checklist sheets [14]. Another important tool for assessing an exam’s reliability is factor analysis, which is used to quantify skills, ensure the components of the OSCE stations are homogeneous, and identify the structure of the exam [15, 16]. An important advantage of the OSCE is the feasibility of assessing the validity of the exam. The most commonly used index for this is Pearson’s correlation, which is a useful tool for assessing the correlation between the OSCE score and the written exam and has been used in many published articles [17–19]. Most published reports have been about the advantages of OSCE as a reliable and valid examination method, but none have focused on the reliability of the indexes used in the assessment of the exam and whether a small difference between them means a single index is sufficient [17, 20].

## Study aims

The aims of this study are as follows:

To obtain a reliability and validity index for the exam.

To evaluate whether a single reliability index is enough to assess the OSCE and to ensure fairness among all participants.

## Results

The reliability for the OSCE was evaluated using Cronbach’s alpha to indicate the stability of the stations on the three exams. The alphas for the three groups were 0.7, 0.8, and 0.9, showing an increase in a linear pattern. Spearman’s rank correlation and R2 coefficient determinants were used to correlate the checklist results with the global score to arrive at an internal consistency score. The correlations were 0.7, 0.7, and 0.8 (*p* < 0.001) for both Cronbach’s alpha and Spearman’s rank correlation, which indicated a strong correlation between the checklist score and global rating on all days of the exam. The R2 coefficient determinants, which were used to examine the linear correlation between the checklist and the global score, were 72, 82, and 78.2 %. Spearman’s rank correlation and the R2 coefficient determinant values did not differ, which indicated good internal consistency. However, it did not increase in the same manner as the Cronbach’s alpha for stability. Spearman’s rank correlation was stable in the first and second group and increased slightly with the third group, with a slight decrease in the R2 coefficient in the last group after a slight increase in the second group (Table 1).

**Table 1:** Reliability measures for the 4th-year OSCE

Day/data group	Gender	Days	Students/h/day	Stability^a^	Internal consistency^b^	*P* value	Internal consistency^c^
First group	Male	2	56/3–3.3 h/day	0.7	0.7	<0.001	0.72 (72 %)
Second group	Female	3	97/3–4 h/day	0.8	0.7	<0.001	0.82 (82 %)
Third group	Male	2	54/3.3–4.3/day	0.9	0.8	<0.001	0.782 (78.2 %)

^a^Cronbach’s alpha

^b^Spearman’s rank correlation

^c^R2 coefficient determinants

**Fig. 1:**
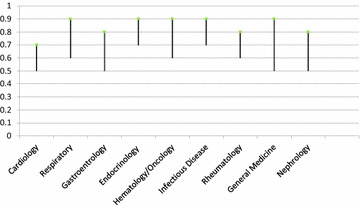
Cronbach’s alpha by OSCE system

**Fig. 2:**
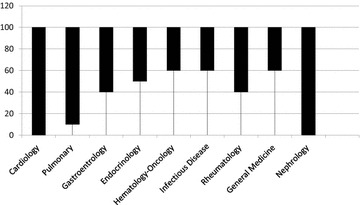
OSCE scores by system

The Cronbach’s alphas for the stations ranged from 0.5 to 0.9. Figure 1 shows the Cronbach’s alpha scores for stations based on the systems. The values were lowest for the nephrology, gastroenterology and cardiology examination stations. The endocrinology and infectious disease stations were the best, followed by hematology–oncology, general medicine and respiratory system stations (Cronbach’s alpha = 0.8–0.9). The other systems fluctuated between high and low alphas (Cronbach’s alpha = 0.6–0.9). The score ranges for each system are shown in Fig. 2 and were calculated based on a total possible score of 100.

**Fig. 3:**
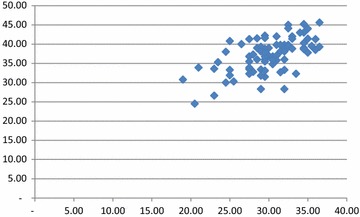
Pearson’s correlations for the exam

The OSCE scores for the students were between 18.7 and 36.9, with a mean of 27.6, a median of 27.9, a standard deviation (SD) of 4.07, a skewness of −0.07 (which is almost 0),and a normal distribution, where the definition of skewness is described as asymmetry from the normal distribution in a set of statistical data. Kurtosis, which is a statistical measure used to describe the distribution of observed data around the mean (2.37), indicated that the curve was flatter than a normal distribution with a wider peak. The probability for extreme values was less than for a normal distribution, and the values had a wider spread around the mean. The OSCE score analysis for the students is shown in detail in Table 2. The reliability of the written exam was 0.79, which is considered very good. The students needed to score at least 60 % on the OSCE and 60 % on the written exam to pass the course. The score analysis for the written exam is shown in detail in Table 3. The lowest score was 18.1 and the highest was 43.1 (out of 50 %) for the 4th-year students, with a mean of 33.6, a median of 33.75, an SD of 4.35, and a relative SD of 12.9. To measure the validity of the exam, we conducted a Pearson’s correlation to compare the results of the OSCE and written exam scores. The correlation was 0.63, which indicated a strong correlation between the OSCE score and the written exam score (Fig. 3). Finally, a factor analysis (with rotated factors) was conducted to ensure that the components of the OSCE stations were homogenous, to identify the structure of the exam that best reflects the exam selection stations, to determine how the exam structure relates to the variables, and to determine if the OSCE assessed the students professional clinical skills. The values of the rotated factors ranged from 0.1 to 0.99. However, most of the stations were between good and very good (Table 4). These results support the validity of the exam.

**Table 2:** Analysis of the 4th-year OSCE scores (total possible = 40)

Statistical parameters	Result
Minimum	18.7
Maximum	36.9
Range	18.2
Count	207
Mean	27.6
Median	27.9
Mode	26.8, 25.5, 30.8, 28.3
Standard deviation	4.07
Variance	16.5
Mid-range	27.8
Quartiles	Quartiles
	Q_1_ → 24.9
	Q_2_ → 27.9
	Q_3_ → 30.8
Interquartile range (IQR)	5.9
Mean absolute deviation	3.30
Root mean square (RMS)	27.9
Std error of mean	0.28
Skewness	−0.07
Kurtosis	2.37
Coefficient of variation	0.14
Relative standard deviation	14.7 %

**Table 3:** Analysis of the 4th-year written exam

Statistical parameters	Results
Minimum	18.125
Maximum	43.125
Range	25
Count	207
Mean	33.6
Median	33.75
Mode	34.37
Standard deviation	4.35
Variance	18.9
Mid-range	30.625
Quartiles	Quartiles
	Q_1_ → 30.62
	Q_2_ → 33.75
	Q_3_ → 36.25
Interquartile range (IQR)	5.625
Mean absolute deviation	3.48
Root mean square (RMS)	33.9
Std error of mean	0.302
Skewness	−0.34
Kurtosis	3.36
Coefficient of variation	0.12
Relative standard deviation	12.93 %

**Table 4:** Factor analysis for the 4th-year results

Stations	Factor 1
V1	0.64
V2	0.547
V3	0.713
V4	0.499
V5	0.694
V6	0.621
V7	0.154
V8	0.39
V9	0.613
V10	0.604
V11	0.675
V12	0.795
V13	0.804
V14	0.684
V15	0.752
V16	0.682
V17	0.991
V18	0.991

## Discussion

This was a pilot study conducted in the Internal Medicine department of Dammam University in 2014. The reliability for the OSCE exam was in the acceptable range in all groups, but there were differences in the results that support our hypothesis that no single reliability index can be considered a perfect tool for assessing the OSCE.^1^ There was no difference between the male and female groups in the exam reliability results, which means that gender does not affect the results. Pearson’s correlation was 0.63, which demonstrates that the OSCE is a valid exam. The number of students who took the exam provided a very good sample size, and the reliability of the OSCE stations was good for all three index measures used. We started with Cronbach’s alpha to measure the stability of the stations. This value increased with each subsequent exam, which may have been because the exam durations increased progressively.^2^ In particular, the third group took longer because of changing the patients secondary to their request and because of the large number of students. As a result, this may have produced a misleading value that is not as reliable, and this is the main disadvantage of Cronbach’s alpha (Table 1) [3, 5, 13]. Spearman’s rank correlation and the R2 coefficient determinants are internal consistency measures and were found to be different from the Cronbach’s alpha results. While there was a progressive increase in Cronbach’s alpha, the Spearman’s rank was stable in the first and second group and increased in the third group, which indicates stronger internal consistency in the last group. The R2 coefficient increased in the second group and then decreased in the third, which may have been because the examiner made the checklist score correspond to the global score in the second group. This was the result of faculty misunderstanding because it was a first time experience.^3^ This issue was managed with feedback after each exam to avoid these mistakes in future exams. The internal consistency and reliability results improved in general, which can be explained by the time effect and the examiner misunderstanding the global score. However, the encouraging point is that the differences between the R2 values were very small. Finally, this study highlighted the deficits in reliability indexes, something that has not been the focus of many studies on the OSCE. It was thus discovered in our study that Cronbach’s alpha is not sufficient for measuring reliability. Adding Spearman’s rank correlation and the R2 coefficient gives more accurate and reliable results, which is fairer to the examinees participating in the examination because it provides the following: better assessment of the students’ clinical skills (history, physical examination, communication skills, and data interpretation) and increased fairness of the exam stations. Our study is one of few that have focused on reliability indexes; to date, three publications have measured the reliability and validity of the OSCE using a maximum of three measures. The first study included factor analysis for a medical course, and the other discussed in detail the use of the OSCE for an internal medicine course, which is a multi-system course. The second study was the first to discuss the effect of exam duration on the reliability index of the OSCE and reported on the effect of different days of the exam on its validity [7, 15, 16].

When we compared the OSCE scores to the written scores, the results were normally distributed with a slight left skew. This indicated that students were performing better than expected and that the exam was a good stimulator for reading. The validity of the exam was measured by Pearson’s correlation, which was strong. We look forward to having very strong validity in the next few years. The results of this study are stimulating and should encourage other clinical departments at Dammam University to use the OSCE in the future. The findings could help internal medicine departments in our institute and in other medical colleges to improve the OSCE station reliability by considering multiple tools to assess the reliability of the stations and not focus solely on one index, especially given the disadvantages of each measurement tool. Compared to other studies reporting the reliability and validity of the OSCE, this is the only report that has focused on the measurement tools and index defects in an internal medicine course. Most of the published reports have concentrated on the reliability and validity of the exam, feedback, and gender differences, which are some of the most important issues for undergraduate students and part of a university’s mission and vision. The OSCE can be a vital teaching tool. This study demonstrated improvement in conducting the OSCE through experience, which was reflected by the increase in the reliability indexes after each exam. This increase occurred over a short period as a first experience for the department of internal medicine. Importantly, although the exam occurred on different days, this did not change the validity of the exam, a result that few studies have reported.

## Limitations

First, this study was conducted on a single department within a single institution and involved only 4th-year medical students who agreed to the new examination format. The students in their final year did not participate due to the potential stress and lack of familiarity with the style of the exam. Second, the examiners were not the same for the duration of the study due to their commitments with clinics and inpatient services. The third limitation is that the topic of management was omitted from the exam, even though it is included in the curriculum. Finally, the distribution of students was dependent on their registration in the university, which resulted in different numbers of students enrolled for each course.

## Conclusion

No single reliability index can be considered as a perfect tool for assessing the OSCE. To solve this issue, there must be at least two to three indexes to ensure the reliability of the exam. Pearson’s correlation is considered a good measure for assessing the validity of OSCE.

Similar studies should be conducted within all clinical departments and at other medical schools to further understand the strengths and weaknesses of the reliability indexes and to identify the number of indexes to be used to ensure the reliability of the exam. Such research can lead to a more reliable and valid OSCE in the future.

## Methods

### Participants

This pilot study was conducted over one semester (February–May) with 207 year four medical students (the first clinical year after they completed and passed all preclinical courses) as per university law, who took the exam in three groups (in March, April, and May, 2014). At the end of the semester, the students took the written exam (control exam), consisting of 80 multiple-choice questions.

### Procedure

Introductory lectures on the OSCE were held for the faculty to explain the stations, the importance of the rubric for the checklist, and the global ratings. An introduction and orientation about the OSCE was also given to each student group on the first day of the course. The blue print for each exam was established. The OSCE had 18 clinical stations (with no repeated stations) and covered history, physical examination, communication skills, and data interpretation. Each station took 7 min to complete. Students were divided into groups as shown in Table 1. The blueprint for each group covered all the systems in internal medicine, including communication skills, cardiology, the respiratory system, gastroenterology, endocrinology, hematology-oncology, nephrology, infectious disease, rheumatology, and general medicine. The exception was neurology, which was covered in a separate course. The exams were conducted for 3–4.3 h/day over 7 days for all three groups. The highest possible score was 100 %; the OSCE exam accounted for 40 %, a continuous assessment accounted for 10 %, and the written exam accounted for 50 %. All 207 students took the clinical and written exams. After each exam, the coordinator of the course met with faculty and students to assess and correct any problems with the OSCE to ensure better reliability in the future and they were confidents with OSCE.

### Ethical considerations

The study was approved by the Institutional Review Board of the University of Dammam (Approval number: IRB-2014-01-317). Informed written consent was obtained from all participants.

### Data analysis

The exam’s reliability, which is defined as the degree to which an assessment tool produces stable and consistent results, was assessed by Cronbach’s alpha, the global rating (clear pass, borderline, or clear fail), and the coefficient of determination R2. Spearman’s rank correlation was used to evaluate the correlation between the checklist and global rating scores. Finally, a factor analysis was used to assess exam homogeneity. At the end of the semester, each student took the written exam (control exam), which was analyzed (mean, median, and mode) separately for each year. The validity, which refers to how well a test measures what it is purported to measure, was measured by Pearson’s correlation. Analyses were conducted for each system to understand any deficits in the courses.

## Footnotes

1 Cronbach's alpha, Spearman’s rank correlation, and R2 coefficient determinants are reliability indexes and none is considered the best single index.

2 Cronbach's alpha is affected by exam duration.

3 The R2 coefficient is affected if there is faculty misunderstanding of the difference between the checklist and global rating.
